# Effect of Oral Iron Supplementation on Cognitive Function among Children and Adolescents in Low- and Middle-Income Countries: A Systematic Review and Meta-Analysis

**DOI:** 10.3390/nu14245332

**Published:** 2022-12-15

**Authors:** Zekun Chen, Huanhuan Yang, Dongqing Wang, Christopher R. Sudfeld, Ai Zhao, Yiqian Xin, Jiawen Carmen Chen, Wafaie W. Fawzi, Yan Xing, Zhihui Li

**Affiliations:** 1 Vanke School of Public Health, Tsinghua University, Beijing 100084, China; 2 Department of Global Health and Population, Department of Nutrition, Harvard T.H. Chan School of Public Health, Harvard University, Boston, MA 02115, USA; 3 Duke Global Health Institute, Duke University, Durham, NC 27705, USA; 4 Department of Population and Public Health Sciences, Keck School of Medicine, University of Southern California, Los Angeles, CA 90089, USA; 5 Department of Pediatrics, Peking University Third Hospital, Beijing 100191, China

**Keywords:** iron supplementation, children and adolescents, cognitive function, meta-analysis

## Abstract

Background: There is abundant evidence showing that iron deficiency is closely linked with delayed brain development, worse school performance, and behavioral abnormalities. However, evidence on the impact of iron supplementation among children and adolescents in low- and middle-income countries (LMICs) has been inconsistent. This study aims to examine the effect of oral iron supplementation on cognitive function among children and adolescents in LMICs. Methods: A systematic review and meta-analysis was conducted to examine the impact of iron supplementation on cognitive function (including intelligence, attention, short-term memory, long-term memory, and school performance) among children and adolescents aged 5 to 19. We searched PubMed, Embase, Web of Science, CINAHL, and references of related articles published from the inception of the databases to 1 May 2022. Random-effects pooled standardized mean difference (SMD) with 95% confidence intervals (CIs) were calculated to estimate the effect of iron supplementation on cognitive function. We also investigated the heterogeneity of the effects using subgroup and meta-regression analyses. This review was registered with PROSPERO (CRD42020179064). Results: Nine studies with 1196 individual participants from five countries were identified and included. Iron had a positive impact on intelligence test scores among children and adolescents (SMD = 0.47, 95% confidence interval [CI]: 0.10, 0.83). Meta-regression showed that the intelligence test scores improved with increasing the iron supplement dose (odds ratio [CI] = 1.02, 95% CI: 1.00, 1.04). There were no significant effects on attention, short-term memory, long-term memory, or school performance. Conclusions: Oral iron intake can improve the intelligence test scores of children and adolescents in LMICs and should be considered for future nutritional interventions.

## 1. Introduction

Children and adolescents aged 5 to 19 years old are going through a period of profound development. Rapid physical, biological, and hormonal changes that lead to the psychosocial, behavioral, and sexual maturation of the individual characterize this life period [1]. Because of the inability of the body to rapidly increase iron reserves when demand increases, iron deficiency often occurs during this period [2]. The Global Burden of Disease (GBD) Study 2019 estimated that 16.83% of children and adolescents aged 5–19 years old globally suffered from iron deficiency; the prevalence in high-income countries, upper-middle-income countries, lower-middle-income countries, and low-income countries was 4.11%, 5.73%, 23.19%, and 22.20%, respectively [3].

As we can see, children and adolescents living in low- and middle-income countries (LMICs) are particularly vulnerable to anemia and iron deficiency [4]. For instance, in Bangladesh, anemia is a public health problem, with 40% of children and adolescents suffering from anemia [5], and up to 60% of school-aged children in Malaysia suffer from varying degrees of anemia [6]. Realizing the importance, growing action has been taken to reduce anemia and iron deficiency among children and adolescents in LMICs. For instance, children and adolescents aged 6–18 years in India were included in the National Nutrition Anemia Prophylaxis Programme (NNAPP) in 2007 [7]. In the same year, Bangladesh published the National Strategy for Anemia Prevention and Control, in which school-age children and non-pregnant women of reproductive age are provided with the recommended dose and frequency of iron-folate (IFA), as well as being screened for severe anemia and provided with appropriate anemia treatment [8]. Moreover, the World Health Organization (WHO) published guidelines for daily iron supplementation in 2016 [9,10], in which daily iron supplementation is recommended as a public health intervention for children aged 60 months and older living in areas where 40% of infants and young children have anemia [9]. Anemia is defined as the reduction of the number and volume of red blood cells in the peripheral blood of the organism, and the reduction of hemoglobin content, which cannot deliver enough oxygen to the peripheral blood tissue. Anemia not only has a negative impact on the health of individuals but also hinders economic and social development, especially in childhood, where it can demonstrate an irreversible effect on the growth and development of children and even affects their ability to work as adults. 

Aside from anemia, iron deficiency is also associated with cognitive dyspraxia [11,12]. Iron is involved in numerous brain physiological processes, including the creation of neuronal myelin sheaths and a variety of neurotransmitters [13]. Moreover, iron is also required for metabolism, neurotransmission, and neurogenesis, and it has an impact on behavior, memory, learning, and sensory systems [14]. Iron deficiency can hinder the growth and development of children and adolescents, leading to decreased anthropometric parameters, cognitive impairments, and even behavioral abnormalities [15,16]. Studies showed that even mild iron deficiency can have profound adverse effects on the cognitive development of individuals [17,18], and the adverse health effects of iron deficiency persist even after the iron status is improved [19]. Iron supplements can help increase the levels of iron in the body, which promotes the production of healthy red blood cells and the delivery of oxygen to body tissues. In turn, this might can improve cognitive function and relieve symptoms such as fatigue and difficulty concentrating. Iron supplementation is widely recommended for the treatment of IDA and anemia. Moreover, oral iron supplementation is the most widely used form of iron supplementation in clinical practice.

The impact of iron supplementation on anemia and iron deficiency among children and adolescents in LMICs has been confirmed; however, evidence on the relationship between iron supplementation and cognitive function has been inconsistent. For example, a study located in India of 120 adolescents showed significant improvements in cognitive function and academic performance in participants who received 100 mg of elemental iron supplementation six days a week for eight months, with or without anemia and iron deficiency [20], whereas others [21,22] reported its ineffectiveness.

Understanding the effects of iron supplementation on cognition among children and adolescents is essential for the appropriate design of targeted policies. This systematic review and meta-analysis aimed to comprehensively analyze the effects of iron supplementation on cognitive outcomes in children and adolescents in LMICs.

## 2. Materials and Methods

### 2.1. The Study Protocol and Search Strategy

This study protocol followed the Cochrane Handbook for Systematic Reviews and Meta-Analysis Protocol (Cochrane Handbook for Systematic Reviews and Meta-Analysis Protocol) (PRISMA-P) [23], and was registered with the International Prospective Register of Systematic Reviews (PROSPERO identifier: CRD42020179064).

Under the direction of a professional librarian, the literature search for this study was performed in PubMed, Embase, Web of Science, and CINAHL. All studies were published before May 1, 2022. The search included relevant medical subject heading terms, keywords, and word variants for children, adolescents, iron supplementation, and LMICs (see Supplementary Text S1 for full search strategies and results). The list of references to related reviews and articles was also checked. Moreover, we contacted the authors of articles via email when the required information to determine eligibility was not reported in the articles to ensure that all necessary data were obtained. 

### 2.2. Selection Criteria

Studies for this meta-analysis were selected based on the following a-priori-defined inclusion criteria:

(1) Randomized controlled trials (RCTs);
(2) Children and adolescents within the age range of 5–19 years;
(3) From LMICs classified by the World Bank according to the year of the study;
(4) Oral iron supplementation where iron was the only micronutrient provided;
(5) The control group could be a placebo or no intervention.

Exclusion criteria:

(1) Interventional studies without appropriate control groups;
(2) Observational studies;
(3) Editorials, reviews, opinions, or review articles were ineligible; however, these articles were reviewed to determine eligible research;
(4) Participants with pregnancy or HIV/AIDS, or lactating.

If study samples overlap in at least two publications or if multiple publications describe the same study aspects, then only the publication with the largest sample was considered.

### 2.3. Data Extraction

Data were extracted using a standardized spreadsheet independently by two reviewers (Yiqian Xin and Jiawen Carmen Chen) on a prespecified form. A third author (Zekun Chen) was consulted when discrepancies occurred. All literature meeting inclusion criteria were carefully read. Standard data extraction tables were used to extract specific information from each study, including the first author, publication date, research location, age at intervention, dose of iron supplementation, intake duration, and the number of people in the intervention group and control group. Additionally, mean and standard deviation (SD) or standard error (SE) were extracted from each study included.

### 2.4. Outcome

The main indicators used to assess cognitive function in this study included intelligence, attention, short-term memory, long-term memory, and school performance (Supplementary Text S2). In order to standardize assessments and increase objectivity, we use mathematical scores to assess school performance. 

### 2.5. Quality Assessment

The quality assessment for each included study was conducted independently by two reviewers (Jiawen Chen and Zekun Chen) and performed using the Cochrane Collaboration’s Risk of Bias (RoB) assessment tool (Review Manager version 5.4) [24]. The assessment of quality characteristics used the following criteria: (1) random sequence generation; (2) allocation concealment; (3) blinding of participants and personnel; (4) blinding of outcome assessment; (5) incomplete outcome data; (6) selective reporting; and (7) other biases. We judged each domain as "low risk of bias," "high risk of bias," or "unclear risk”. We considered an RCT to have a low risk of bias if it was judged to have a low risk of bias in all domains. We considered an RCT to have a high risk of bias if it was judged to have a high risk of bias in at least one domain. We considered an RCT to have unclear risk if an RCT had unclear risk in at least one domain.

### 2.6. Statistical Analysis

Forest plots were generated for pooled analysis, and a standardized mean difference (SMD) was reported in the analysis. The heterogeneity among results was analyzed by the Q test. If the Q test obeyed the chi-square distribution and *p* >0.1, then it was judged to have no heterogeneity. *I*^2^ statistic, which can be used to quantitatively represent the between-study heterogeneity, was calculated in this study. A fixed-effect model was adopted if *I*^2^ < 50%; otherwise, the random-effects model was performed. Subgroup analyses on intelligence by sex, age, dose of iron exposure, supplementation duration of iron, and baseline anemia status of the study participants were conducted according to the different characteristics of the studies (the number of studies for other outcomes was not sufficient for subgroup analysis). In each outcome, sensitivity analysis was employed to investigate the influence of a single trial on the overall effect as estimated by omitting one study in each turn. Publication bias was assessed with Egger’s linear regression test [25]. Where the SD was unavailable but the SE was available, the SD was calculated from the SE (SD = SE × $\sqrt{N}$). (Supplementary Text S2). All statistical analyses were performed with Review Manager 5.4 (Review Manager 2020) and STATA (version 17; Stata Corp., College Station, TX, USA). *p* < 0.05 was considered statistically significant.

## 3. Results

### 3.1. Document Retrieval Results

**Figure 1:**
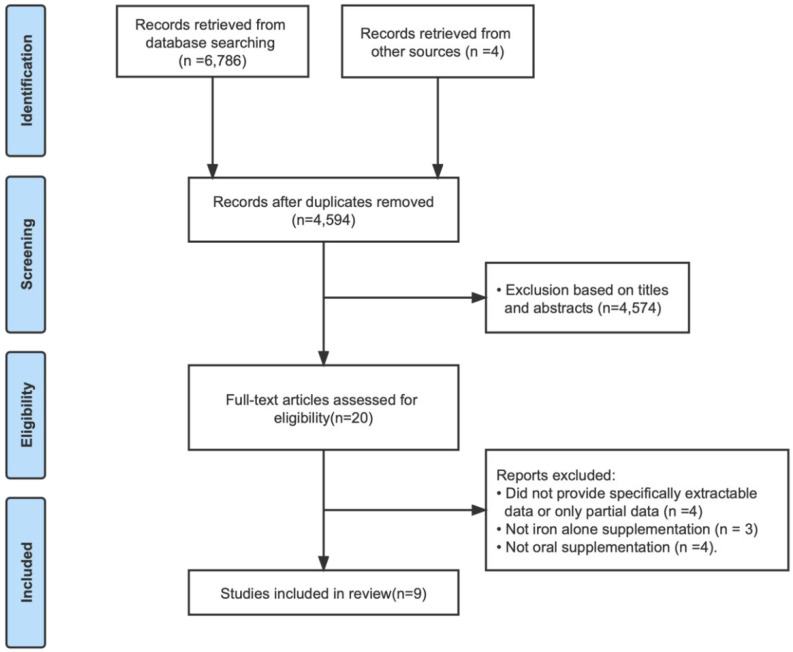
Flowchart of Search Strategy.

Figure 1 displays the flowchart of this study. Specifically, the search strategy identified a total of 4594 articles after removing duplicates, of which 4574 were excluded based on the contents of the title and abstract. After this, we retrieved 20 full-text papers for further consideration. Among them, four records were excluded for not providing sufficiently extractable data; three were excluded because they were not supplementation with iron alone; and four were excluded for not being oral supplementation. Ultimately, nine RCTs fulfilled the eligibility criteria and were included in the meta-analysis [20,26,27,28,29,30,31,32].

### 3.2. Basic Features Included in The Study

Table 1 presents a summary of the characteristic of 1196 participants across the included studies. All the studies were RCTs conducted in LMICs and published in 1988 and beyond, with the latest published in 2014 [20,26,27,29,30,31]. Among these studies, eight were carried out in Asia (four in India, one in Indonesia, one in Iran, and two in Thailand) and one in Africa (South Africa). The age of children and adolescents at the time of intervention was 6–18 years old. The median intervention duration was 4.9 months (varied from 3 months to 8.5 months). Some of the trials had two arms providing different doses of the supplement regimen. In these cases, we combined the study arms for the overall comparison and included the disaggregated information in the subgroup analyses (Supplementary Text S3). 

### 3.3. Outcomes

**Figure 2:**
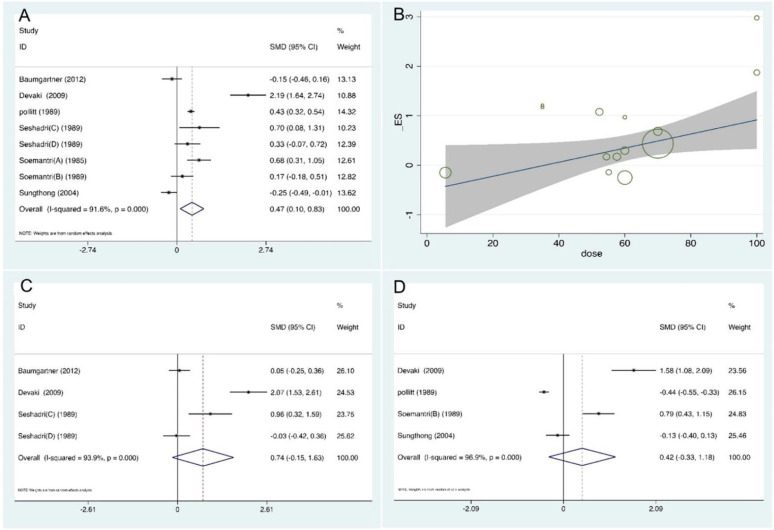
Summary analysis of cognitive function (**A**) Forest plot of the effect of iron supplementation on intelligence in children and adolescents. (**B**) Meta-regression analysis on the dose of the effect of iron supplementation on intelligence in children and adolescents. (**C**) Forest plot of the effect of iron supplementation on short-term memory in children and adolescents. (**D**) Forest plot of the effect of iron supplementation on school performance in children and adolescents [20,26,27,29,30,31,32].

Eight studies investigated the effect of iron supplementation on the intelligence development of children and adolescents [20,26,27,29,30,31]. The intervention group had a significant increase in intelligence after taking the iron supplementation compared to the control group (SMD = 0.47, 95% confidence interval [CI]: 0.10, 0.83, *p* = 0.012, *I*^2^ = 91.6%) (See Figure 2A). Meta-regression showed that the intelligence test scores improved with increasing iron supplement dose (OR = 1.02, 95% CI: 1.00, 1.04, *p* = 0.020) (Figure 2B).

Three studies (Rezaeian, Seshadri (C), and Seshadri (D)) [28,29] reported the effects of iron supplementation on attention in children and adolescents. The result of the pooled analysis revealed that the intervention group had a significant improvement in attention after iron supplementation (SMD = 11.38, 95% CI: 3.94, 18.82, *p* < 0.001, *I*^2^ = 99.5%). However, in the sensitivity analysis, after deleting the Rezaeian et al. study [28], the result was not consistent with the pooled analyses, so we analyzed the following sections of this paper excluding the study of Rezaeian et al. After that, the result of the pooled analysis showed that there was no statistically significant difference between groups receiving iron supplementation and a placebo (SMD = 0.27, 95% CI: −0.09, 0.63, *p* = 0.143, *I*^2^ = 0.0%) (available in Supplementary Figure S1, Supplementary Figure S2). The domain of short-term memory was evaluated by four trials [20,26,29]. Pooled analysis (Figure 2C) showed that there was no evidence of a statistically significant difference in the iron supplementation group compared with the control group (SMD = 0.74, 95% CI: −0.15, 1.63, *p* = 0.104, *I*^2^ = 93.9%). The relationship between iron supplementation and long-term memory in children and adolescents was demonstrated in two trials [20,26]. There was no significant difference between groups (SMD = 0.94, 95% CI: −0.90, 2.77, *p* = 0.315, *I*^2^ = 96.5%). Four studies analyzed an association between iron supplementation and school performance in children and adolescents [20,27,31,32]. The pooled results (Figure 2D) report that iron supplementation had no significant effect on the development of school performance in children and adolescents. (SMD = 0.42, 95% CI: −0.33, 1.18, *p* = 0.275, *I*^2^ = 96.9%).

### 3.4. Subgroup Comparisons

To examine the different effects of iron among various groups, we performed subgroup analyses on the outcomes of intelligence. Table 2 shows the results of the subgroup analyses by sex, age, dose, duration, and iron status on intelligence. Iron supplementation had a significant beneficial effect on intelligence for participants older than 11 years (SMD = 1.18, 95% CI: 0.33, 2.04, *p* = 0.007, *I*^2^ = 89.4%), and daily supplementation dose ≥60 mg/day (SMD = 0.91, 95% CI: 0.38, 1.45, *p* = 0.001, *I*^2^ = 93.8%). Moreover, intelligence was also elevated significantly when iron supplementation duration was ≥4 months (SMD = 0.81, 95% CI: 0.38, 1.25, *p <* 0.001, *I*^2^ = 92.0%). In addition, iron status was divided into the anemia group (SMD = 1.01, 95% CI: 0.34, 1.68, *p* = 0.003, *I*^2^ = 88.6%), and the non-anemia group (SMD = 0.68, 95% CI: 0.17, 1.18, *p* = 0.009, *I*^2^ = 82.3%), and both showed significant effects. 

### 3.5. Methodological Quality, Publication Bias, and Sensitivity Analysis

Each included study was assessed according to the RoB, and Supplementary Figure S3 presents the RoB graph and the RoB summary. Overall, five of the studies were categorized as having a low risk of bias, two as having an unclear risk, and two as having a high risk of bias. Additionally, there was no evidence of a significant publication bias in this study, confirmed by the Egger’s test (Intelligence test score: *p* = 0.737; short-term memory: *p* = 0.231; school performance: *p* = 0.051). In the leave-one-out sensitivity analyses, the impact of each trial was discussed, testing whether deleting each trial sequentially would significantly change the pooled results of the meta-analysis (available in Supplementary Figure S2). Sensitivity analyses of each individually deleted study showed that none of the studies alone significantly changed the pooled results of our outcomes except attention (see Supplementary Figure S2 for details).

## 4. Discussion

This study suggests that oral iron supplementation is beneficial for the intelligence development of children and adolescents from LMICs and that the beneficial effect of oral iron supplementation on intelligence increases with higher doses of iron supplementation. Furthermore, meta-regression analyses revealed that the positive effect of oral iron supplementation on intelligence increased when the iron supplementation dose was elevated. In addition, iron supplementation can improve the intelligence test score of children and adolescents whether they are anemic or not. 

This study found a positive effect of iron intake on intelligence test scores in children and adolescents, which is consistent with the findings of the present study. A meta-analysis included 14 RCTs of children aged over 6 years old, adolescents, and women [33] noted that iron supplementation can improve the intelligence score in anemic children and intelligence quotient increased by 2.5 points (95% CI: 1.24,3.76), but has no effect on memory, psychomotor skills, or academic performance. In a meta-analysis of 17 RCTs, Sachdev et al. [34] examined the effects of iron on mental and motor development in infants and children and showed that iron supplementation has a minor but positive effect on mental development. This impact is notably stronger in intelligence tests performed above the age of seven and in children who had anemia or an iron deficiency at baseline. Iron deficiency has been found to cause significant changes in striatal neural metabolites in rodent models that avoid significant growth restriction [35]. The striatum is an integral part of the basal ganglion; the network of the striatum is associated with higher-order cognitive and emotional processes, motivated behavior, positive emotion, reward-related processing, and motor functions [36,37]. It is worth noting that although this study did not find a negative effect of iron supplementation dose on intelligence, previous studies have suggested that high doses of iron supplements may cause potentially harmful effects, and Sungthong et al. [32] reported that children who supplemented daily had lower increases in IQ scores than children who supplemented weekly. There are two possible explanations suggested by the authors. The first mechanism is the direct effect of high iron stores on cognitive function, which may lead to oxidative stress. The other is an indirect effect on cognitive function due to reduced absorption or redistribution of other nutrients after iron supplementation.

This study demonstrated that iron supplementation had no significant impact on memory and attention. The previous studies reported inconsistent findings regarding the attention and memory domain. Baumgartner et al. [26] reported that the beneficial effect of iron supplementation on long-term memory only exists in IDA children, whereas no impacts have appeared in nonanemic children. A meta-analysis including 50 studies [38] showed that iron status did not directly impact attention, whereas Falkingham [33] et al. found that iron supplementation improved attention in adolescents and women over 8–17 weeks regardless of baseline iron status. Moreover, this study found that iron supplements have no overt effects on school performance in children and adolescents. It might be because school performance is influenced by many sources including family, social environment, genetics, and so on. In addition, school performance necessitates a significantly longer intervention period than the time required to replenish iron levels. Even if iron concentrations have improved, performance may take another period to improve. This is especially important when discussing school performance since the iron state during learning might differ from the iron level during knowledge retrieval or performance evaluation. This may cause an undetectable effect of iron supplementation in such trials [33].

Subgroup analysis revealed a significant improvement in intelligence scores after oral iron supplementation when the supplementation duration of supplementation was ≥4 months. This might be because the turnover of red blood cells requires 90–120 days [39], in addition, Youdim et al. [40] found that in the rat model liver iron was replenished more rapidly than brain iron. This may also reflect that it takes longer for the effects of iron supplementation to reach the brain than the liver, which suggests that we need to pay attention to the duration of iron supplementation. Previously, the policy of iron supplementation tended to be skewed toward infants and young children, whereas adolescents were often overlooked [9]. This study showed that iron supplementation significantly promoted intelligence test scores in adolescents >11 years of age, suggesting that iron supplementation is also important for adolescents. It is similar to a previous study that reported that iron supplementation has a positive effect on mental development in older children (>8 years old), but not significantly in younger children [34]. In addition, iron supplementation could improve the intelligence scores, whether the study participants were anemic or not. This result indicates that iron supplementation may not only benefit the intelligence of anemic children and adolescents but also that of non-anemic children and adolescents. 

This study has its advantages and limitations. To the best of our knowledge, this is the first systematic review focused on iron supplementation’s effects on cognition among children and adolescents aged 5–19 years in LMICs. In addition, we only included RCTs involving iron treatments to increase the homogeneity of the intervention and facilitate meta-analyses. However, there are some limitations. First, varied methodologies were used to measure cognitive function in the included studies, which might lead to heterogeneity. Although we used standardized measures and conducted subgroup analyses, there were still some shortcomings; for example, the baseline iron status cannot be measured and differentiated. Therefore, caution should be exercised in interpreting the results. Secondly, some of the included studies were limited by short intervention periods and small sample sizes. Long-term intervention might be more probable to show the effects of oral iron supplementation on cognitive function in children and adolescents. Finally, we did not conduct subgroup analyses to explore the potential distinctive effects of daily and intermittent iron supplementation, as well as in terms of attention, short-term memory, long-term memory, and school performance, because the number of studies involved was insufficient for subgroup analyses.

## 5. Conclusions

Our results suggest that oral iron intake can improve the cognitive development of children and adolescents living in LMICs. This study justifies the guideline of the WHO, which recommends non-discriminatory iron supplementation for children and adolescents to prevent iron deficiency and anemia in countries where more than 40% of infants and young children have anemia. Moreover, the public health implications of iron should be evaluated in a more comprehensive manner when it comes to iron interventions. Long-term prospective observational studies are needed to evaluate the effects of iron on long-term intellectual development.

## Figures and Tables

**Table 1:** Summary of Eligible Studies.

Author	Country	Age at Intervention, Years	Intervention Group	Control Group	Duration of Intervention	Outcomes
Baumgartner [26], 2012	South Africa	6–11	50 mg*4/wk FeSO_4_ (*n* = 81)	Placebo (*n* = 80)	8.5 months	KABC sequential processing: Triangles, Hand movement, Atlantis Delayed
Devaki [20], 2009	India	15–18	100 mg*6/wk EFe (*n* = 30)	Control placebo (*n* = 30)	8 months	WAIS, Short term memory, long term memory, Scholastic performance test
Pollitt [27], 1989	Thailand	9–11	FeSO_4_ 50 mg/d for 2 weeks and FeSO_4_ 100 mg/d for 14 weeks (*n* = 5)	Placebo (*n* = 4)	4 months	IQ, Mathematics
Rezaeian [28], 2014	Iran	14–18	50 mg*2/wk FeSO_4_ (*n* = 100)	Control group (*n* = 100)	16 weeks	Attention score
Seshadri (C) [29], 1989	India	8–15	(1) 40 mg/d EFe (*n* = 16) (2) 30 mg/d EFe (*n* = 16)	Placebo (*n* = 16)	4 months	Mazes, Digit span, Visual recall
Seshadri (D) [29], 1989	India	8–15	60 mg/d EFe (*n* = 65)	Placebo (*n* = 65)	8 months	Mazes, Digit span, Visual recall
Soemantri (A) [30], 1985	Indonesia	Average 9.5	(1) anemia: 10 mg/kg/d FeSO_4_ (*n* = 43) (2) Non-anemia: 10 mg/kg/d FeSO_4_ (*n* = 16)	(1) anemia placebo (*n* = 35) (2) non-anemia placebo (*n* = 25)	3 mouths	IQ
Soemantri (B) [31], 1989	India	8.1–11.6	2 mg/kg/d EFe (*n* = 37)	Placebo (*n* = 35)	3 months	IQ, math scores
Sungthong [32], 2004	Thailand	6–13	(1) 300 mg/d FeSO_4_ (*n* = 140); (2) 300 mg/wk. FeSO_4_ (*n* = 134)	Placebo (*n* = 123)	16 weeks	IQ, mathematics

Abbreviations: EFe: elemental iron; FeSO_4_: ferrous sulfate; IQ: intelligence quotient; WAIS: Wechsler adult intelligence scale; KABC: Kaufman assessment battery for children; wk: week; d: day; mg: milligram; kg: kilogram.

**Table 2:** Subgroup Analyses of the Effects of Iron Supplementation on Intelligence in Children and Adolescents.

Subgroup Analyses	Heterogeneity Test			Pooled SMD Values (95% CI)	Pooled SMD Values’ Tests Statistic	
	q	d.f.	*I* ^2^		Z	*p*
**Sex**						
Whole population	116.61	8	93.1%	0.67 (0.12, 1.22)	2.38	0.017
Male	2.83	2	29.3%	0.64 (0.14, 1.13)	2.53	0.011
Female	1.64	1	39.1%	0.50 (−0.11, 1.11)	1.62	0.106
**Age, year**						
≤11	47.32	6	87.3%	0.29 (−0.03, 0.61)	1.75	0.08
>11	56.39	6	89.4%	1.18 (0.33, 2.04)	2.7	0.007
**Dose, mg/day**						
<60	23.98	6	75.0%	0.38 (−0.06, 0.81)	1.71	0.088
≥60	97.44	6	93.8%	0.91 (0.38, 1.45)	3.37	0.001
**Duration, month**						
<4	12.24	3	75.5%	0.34 (−0.19, 0.86)	1.26	0.208
≥4	112.71	9	92.0%	0.81 (0.38, 1.25)	3.66	<0.001
**Iron status**						
Anemia	43.98	5	88.6%	1.01 (0.34, 1.68)	2.97	0.003
Non-anemia	28.26	5	82.3%	0.68 (0.17, 1.18)	2.63	0.009

Abbreviation: d.f., degree of freedom.

## Data Availability

Not applicable.

## Supplementary Materials

The following supporting information can be downloaded at: https://www.mdpi.com/article/10.3390/nu14245332/s1, Text S1: Search strategies; Text S2: Definition of cognitive function indicators; Text S3: Data conversion and combine the study arms; Figure S1: Forest plot of the effect of iron supplementation on attention in children and adolescents; Figure S2. Sensitive analysis of cognitive function; Figure S3: Risk of bias of included studies [20,26,27,29,30,31,32,34,41].
