# Supplementary material for: Effect of Oral Iron Supplementation on Cognitive Function among Children and Adolescents in Low- and Middle-Income Countries: A Systematic Review and Meta-Analysis
Source: Nutrients. 2022 Dec 15;14(24):5332. doi: 10.3390/nu14245332 (PMC9783508; doi:10.3390/nu14245332)
Supplement: Supplementary file 1 [file nutrients-14-05332-s001.zip › nutrients-2018498-supplementary.pdf]

Supplementary Appendix

Table of Contents

Content	Page
**Supplementary Text S1.** Search strategies	2
**Supplementary Text S2.** Definition of cognitive function indicators	11
**Supplementary Text S3.** Data conversion and combine the study arms	12
**Supplementary Figure S1.** Forest plot of the effect of iron supplementation on attention in children and adolescents	13
**Supplementary Figure S2.** Sensitive analysis of cognitive function	14
**Supplementary Figure S3.** Risk of bias of included studies.	15

Supplementary Text S1. Search strategies

PubMed(n=1,659)

("Iron"[Mesh] OR "Ferric Compounds"[Mesh] OR "Ferrous Compounds"[Mesh] OR iron[tiab] OR "Ferric"[tiab] OR "Ferrous"[tiab] OR "Ferric Compounds"[tiab] OR " Ferrous Compounds"[tiab])

AND

("Adolescent"[Mesh] OR adolescence[tiab] OR adolescent[tiab] OR adolescents[tiab] OR boy[tiab] OR boys[tiab] OR girl[tiab] OR girls[tiab] OR pre pubert*[tiab] OR pre teen*[tiab] OR preadolescen*[tiab] OR prepubert*[tiab] OR prepubescen*[tiab] OR preteen*[tiab] OR puberty[tiab] OR pubescen*[tiab] OR school age*[tiab] OR schoolage*[tiab] OR teen[tiab] OR teenage*[tiab] OR teens[tiab] OR age 5[tiab] OR age 6[tiab] OR age 7[tiab] OR age 8[tiab] OR age 9[tiab] OR age 10[tiab] OR age 11[tiab] OR age 12[tiab] OR age 13[tiab] OR age 14[tiab] OR age 15[tiab] OR age 16[tiab] OR age 17[tiab] OR age 18[tiab] OR age 19[tiab] OR aged 5[tiab] OR aged 6[tiab] OR aged 7[tiab] OR aged 8[tiab] OR aged 9[tiab] OR aged 10[tiab] OR aged 11[tiab] OR aged 12[tiab] OR aged 13[tiab] OR aged 14[tiab] OR aged 15[tiab] OR aged 16[tiab] OR aged 17[tiab] OR aged 18[tiab] OR aged 19[tiab] OR 5 years old[tiab] OR 6 years old[tiab] OR 7 years old[tiab] OR 8 years old[tiab] OR 9 years old[tiab] OR 10 years old[tiab] OR 11 years old[tiab] OR 12 years old[tiab] OR 13 years old[tiab] OR 14 years old[tiab] OR 15 years old[tiab] OR 16 years old[tiab] OR 17 years old[tiab] OR 18 years old[tiab] OR 19 years old[tiab] OR 5 years of age[tiab] OR 6 years of age[tiab] OR 7 years of age[tiab] OR 8 years of age[tiab] OR 9 years of age[tiab] OR 10 years of age[tiab] OR 11 years of age[tiab] OR 12 years of age[tiab] OR 13 years of age[tiab] OR 14 years of age[tiab] OR 15 years of age[tiab] OR 16 years of age[tiab] OR 17 years of age[tiab] OR 18 years of age[tiab] OR 19 years of age[tiab] OR child[tiab] OR children[tiab] OR student[tiab] "child"[MeSH] OR "students"[MeSH] OR child[tiab] OR children[tiab] OR schoolchildren[tiab] OR kids[tiab] OR kid[tiab] OR student*[tiab] OR teen*[tiab] OR adolescen*[tiab] OR preteen*[tiab])

AND

("Developing Countries"[MeSH] OR developing countr*[tiab] OR developing nation*[tiab] OR less developed countr*[tiab] OR less developed nation*[tiab] OR least developed countr*[tiab] OR least developed nation*[tiab] OR third world nation*[tiab] OR third world countr*[tiab] OR under developed nation*[tiab] OR underdeveloped nation*[tiab] OR under developed countr*[tiab] OR underdeveloped countr*[tiab] OR middle income countr*[tiab] OR middle income nation*[tiab] OR low income countr*[tiab] OR low income nation*[tiab] OR poor countr*[tiab] OR poor nation*[tiab] OR lmic[tiab] OR lmics[tiab] OR "Africa"[MeSH] OR "Asia"[MeSH] OR "South America"[MeSH] OR "Latin America"[MeSH] OR "Central America"[MeSH] OR africa[tiab] OR asia[tiab] OR south america*[tiab] OR latin america*[tiab] OR central america*[tiab] OR Afghanistan*[tiab] OR Albania*[tiab] OR Algeria*[tiab] OR Samoa*[tiab] OR Angola*[tiab] OR Armenia*[tiab] OR Azerbaijan*[tiab] OR Bangladesh*[tiab] OR Bengali[tiab] OR Belarus*[tiab] OR Belize[tiab] OR Benin[tiab] OR Bhutan*[tiab] OR Bolivia*[tiab] OR Bosnia*[tiab] OR Herzegovina*[tiab] OR Botswana*[tiab] OR Brazil*[tiab] OR Bulgaria*[tiab] OR Burkina Faso[tiab] OR Burundi*[tiab] OR Cabo Verd*[tiab] OR Cape Verd*[tiab] OR Cambodia*[tiab] OR Cameroon*[tiab] OR Central African*[tiab] OR Chad*[tiab] OR China[tiab] OR Chinese[tiab] OR Colombia*[tiab] OR Comoros[tiab] OR Congo[tiab] OR Cook Islands[tiab] OR Costa Rica*[tiab] OR Cote d'Ivoire[tiab] OR Ivory Coast[tiab] OR

Cuba[tiab] OR Cuban[tiab] OR Djibouti[tiab] OR Dominica*[tiab] OR Ecuador[tiab] OR Egypt[tiab] OR El Salvador*[tiab] OR Eritrea*[tiab] OR Ethiopia*[tiab] OR Falkland Islands[tiab] OR Fiji*[tiab] OR Gabon*[tiab] OR Gambia*[tiab] OR Georgia*[tiab] OR Ghana*[tiab] OR Grenada*[tiab] OR Guadeloupe[tiab] OR Guatemala*[tiab] OR Guian*[tiab] OR Guinea*[tiab] OR Guyan*[tiab] OR Haiti*[tiab] OR Hondura*[tiab] OR India[tiab] OR Indian*[tiab] OR Indonesia*[tiab] OR Iran*[tiab] OR Iraq*[tiab] OR Jamaica*[tiab] OR Jordan*[tiab] OR Kazakh*[tiab] OR Kenya*[tiab] OR Kiribati[tiab] OR People's Republic of Korea[tiab] OR North Korea[tiab] OR Kosovo[tiab] OR Kosovar*[tiab] OR Kyrgyz*[tiab] OR Lao[tiab] OR Laos[tiab] OR Laotian*[tiab] OR Lebanon[tiab] OR Lebanes*[tiab] OR Lesotho[tiab] OR Liberia*[tiab] OR Libya*[tiab] OR Macedonia*[tiab] OR Madagascar*[tiab] OR Malawi*[tiab] OR Malvinas[tiab] OR Malaysia*[tiab] OR Maldives[tiab] OR Mali[tiab] OR Marshall Island*[tiab] OR Mauritania*[tiab] OR Mauriti*[tiab] OR Mayotte[tiab] OR Mexico[MeSH] OR Mexican*[tiab] OR Micronesia*[tiab] OR Moldova*[tiab] OR Mongolia*[tiab] OR Montenegr*[tiab] OR Montserrat[tiab] OR Morocc*[tiab] OR Mozambique[tiab] OR Myanmar[tiab] OR Burmese*[tiab] OR Burma[tiab] OR Namibia*[tiab] OR Nauru[tiab] OR Nepal*[tiab] OR Netherlands Antilles[tiab] OR Nicaragua*[tiab] OR Niger*[tiab] OR Niue[tiab] OR Pakistan*[tiab] OR Paraguay*[tiab] OR Peru*[tiab] OR Philippin*[tiab] OR Pitcairn[tiab] OR Romania*[tiab] OR Rwanda*[tiab] OR Sao Tome[tiab] OR Principe[tiab] OR Senegal*[tiab] OR Serbia*[tiab] OR Sierra Leone*[tiab] OR Solomon Island*[tiab] OR Somalia*[tiab] OR South Africa*[tiab] OR Sri Lanka[tiab] OR St Helena[tiab] OR Saint Helena[tiab] OR St Lucia[tiab] OR Saint Lucia[tiab] OR St Vincent[tiab] OR Saint Vincent[tiab] OR Grenad*[tiab] OR Sudan*[tiab] OR Suriname*[tiab] OR Swaziland*[tiab] OR Eswatini*[tiab] OR Syria*[tiab] OR Tajik*[tiab] OR Tanzania*[tiab] OR Thai*[tiab] OR Timor*[tiab] OR Togo*[tiab] OR Tokelau[tiab] OR Tonga*[tiab] OR Tunisia*[tiab] OR Turkey[tiab] OR Turkish[tiab] OR Turkmen*[tiab] OR Tuvalu*[tiab] OR Uganda*[tiab] OR Ukrain*[tiab] OR Uzbeki*[tiab] OR Vanuatu*[tiab] OR Venezuela*[tiab] OR Vietnam*[tiab] OR Viet nam*[tiab] OR West Bank[tiab] OR Gaza*[tiab] OR Palestin*[tiab] OR Wallis and Futuna[tiab] OR Yemen*[tiab] OR Zambia*[tiab] OR Zimbabw*[tiab] OR Western Sahara[tiab] OR Argentin*[tiab])
AND
("controlled clinical trial" [pt] OR "randomized controlled trial" [pt] OR "clinical trial" [pt] OR "Follow Up" [tiab] OR random*[tiab] OR trial* [tiab])

Embase(n=2,612)
('Iron'/exp OR 'Ferric'/exp OR 'Ferrous'/exp OR 'Ferric Compounds'/exp OR 'Ferrous Compounds'/exp OR iron:ab,ti,kw OR 'Ferric':ab,ti,kw OR 'Ferrous':ab,ti,kw OR 'Ferric Compounds':ab,ti,kw OR 'Ferrous Compounds':ab,ti,kw)
AND
('Adolescent'/exp OR adolescence:ab,ti,kw OR adolescent:ab,ti,kw OR adolescents:ab,ti,kw OR boy:ab,ti,kw OR boys:ab,ti,kw OR girl:ab,ti,kw OR girls:ab,ti,kw OR pre pubert*:ab,ti,kw OR pre teen*:ab,ti,kw OR preadolescen*:ab,ti,kw OR prepubert*:ab,ti,kw OR prepubescen*:ab,ti,kw OR preteen*:ab,ti,kw OR puberty:ab,ti,kw OR pubescen*:ab,ti,kw OR school age*:ab,ti,kw OR schoolage*:ab,ti,kw OR teen:ab,ti,kw OR teenage*:ab,ti,kw OR teens:ab,ti,kw OR 'age 5':ab,ti,kw OR 'age 6':ab,ti,kw OR 'age 7':ab,ti,kw OR 'age 8':ab,ti,kw OR 'age 9':ab,ti,kw OR 'age 10':ab,ti,kw OR 'age 11':ab,ti,kw OR 'age 12':ab,ti,kw OR 'age

13':ab,ti,kw OR 'age 14':ab,ti,kw OR 'age 15':ab,ti,kw OR 'age 16':ab,ti,kw OR 'age 17':ab,ti,kw OR 'age 18':ab,ti,kw OR 'age 19':ab,ti,kw OR 'aged 5':ab,ti,kw OR 'aged 6':ab,ti,kw OR 'aged 7':ab,ti,kw OR 'aged 8':ab,ti,kw OR 'aged 9':ab,ti,kw OR 'aged 10':ab,ti,kw OR 'aged 11':ab,ti,kw OR 'aged 12':ab,ti,kw OR 'aged 13':ab,ti,kw OR 'aged 14':ab,ti,kw OR 'aged 15':ab,ti,kw OR 'aged 16':ab,ti,kw OR 'aged 17':ab,ti,kw OR 'aged 18':ab,ti,kw OR 'aged 19':ab,ti,kw OR '5 years old':ab,ti,kw OR '6 years old':ab,ti,kw OR '7 years old':ab,ti,kw OR '8 years old':ab,ti,kw OR '9 years old':ab,ti,kw OR '10 years old':ab,ti,kw OR '11 years old':ab,ti,kw OR '12 years old':ab,ti,kw OR '13 years old':ab,ti,kw OR '14 years old':ab,ti,kw OR '15 years old':ab,ti,kw OR '16 years old':ab,ti,kw OR '17 years old':ab,ti,kw OR '18 years old':ab,ti,kw OR '19 years old':ab,ti,kw OR '10 years of age':ab,ti,kw OR '11 years of age':ab,ti,kw OR '12 years of age':ab,ti,kw OR '13 years of age':ab,ti,kw OR '14 years of age':ab,ti,kw OR '15 years of age':ab,ti,kw OR '16 years of age':ab,ti,kw OR '17 years of age':ab,ti,kw OR '18 years of age':ab,ti,kw OR '19 years of age':ab,ti,kw OR child:ab,ti,kw OR children:ab,ti,kw OR student:ab,ti,kw 'child'/exp OR 'students'/exp OR child:ab,ti,kw OR children:ab,ti,kw OR schoolchildren:ab,ti,kw OR kids:ab,ti,kw OR kid:ab,ti,kw OR student*:ab,ti,kw OR teen*:ab,ti,kw OR adolescen*:ab,ti,kw OR preteen*:ab,ti,kw)

AND

('developing country'/exp OR @developing countr*@ab,ti,kw OR @developing nation*@ab,ti,kw OR @less developed countr*@ab,ti,kw OR @less developed nation*@ab,ti,kw OR @least developed countr*@ab,ti,kw OR @least developed nation*@ab,ti,kw OR @third world nation*@ab,ti,kw OR @third world countr*@ab,ti,kw OR @under developed nation*@ab,ti,kw OR @underdeveloped nation*@ab,ti,kw OR @under developed countr*@ab,ti,kw OR @underdeveloped countr*@ab,ti,kw OR 'low income country'/exp OR 'middle income country'/exp OR @middle income countr*@ab,ti,kw OR @middle income nation*@ab,ti,kw OR @low income countr*@ab,ti,kw OR @low income nation*@ab,ti,kw OR @poor countr*@ab,ti,kw OR @poor nation*@ab,ti,kw OR lmic:ab,ti,kw OR lmics:ab,ti,kw OR 'Africa'/exp OR 'African'/exp OR 'Asia'/exp OR 'Asian'/exp OR 'South America'/exp OR 'South American'/exp OR 'Central America'/exp OR 'Central American'/exp OR 'Caribbean Islands'/exp OR africa:ab,ti,kw OR asia:ab,ti,kw OR @south america*@ab,ti,kw OR @latin america*@ab,ti,kw OR @central america*@ab,ti,kw OR Afghanistan*:ab,ti,kw OR 'Albania'/exp OR Albania*:ab,ti,kw OR Algeria*:ab,ti,kw OR 'Samoa'/exp OR 'Samoan Islands'/exp OR 'American Samoa'/exp OR 'Pacific islands'/exp OR 'Atlantic islands'/exp OR Samoa*:ab,ti,kw OR Angola*:ab,ti,kw OR Armenia*:ab,ti,kw OR Azerbaijan*:ab,ti,kw OR Bangladesh*:ab,ti,kw OR Bengali:ab,ti,kw OR Belarus*:ab,ti,kw OR Belize:ab,ti,kw OR Benin:ab,ti,kw OR Bhutan*:ab,ti,kw OR Bolivia*:ab,ti,kw OR 'Bosnia and Herzegovina'/exp OR Bosnia*:ab,ti,kw OR Herzegovina*:ab,ti,kw OR Botswana*:ab,ti,kw OR Brazil*:ab,ti,kw OR 'Bulgaria'/exp OR Bulgaria*:ab,ti,kw OR @Burkina Faso@ab,ti,kw OR Burundi*:ab,ti,kw OR @Cabo Verd*@ab,ti,kw OR @Cape Verd*@ab,ti,kw OR Cambodia*:ab,ti,kw OR Cameroon*:ab,ti,kw OR @Central African*@ab,ti,kw OR Chad*:ab,ti,kw OR China:ab,ti,kw OR Chinese:ab,ti,kw OR Colombia*:ab,ti,kw OR Comoros:ab,ti,kw OR Congo:ab,ti,kw OR @Cook Islands@ab,ti,kw OR @Costa Rica*@ab,ti,kw OR 'cote d`ivoire':ab,ti,kw OR @Ivory Coast@ab,ti,kw OR 'Cuban'/exp OR Cuba:ab,ti,kw OR Cuban:ab,ti,kw OR Djibouti:ab,ti,kw OR Dominica*:ab,ti,kw OR Ecuador:ab,ti,kw OR Egypt:ab,ti,kw OR @El Salvador*@ab,ti,kw OR Eritrea*:ab,ti,kw OR Ethiopia*:ab,ti,kw OR 'Falkland Islands (Malvinas)'/exp OR @Falkland Islands@ab,ti,kw OR Fiji*:ab,ti,kw OR Gabon*:ab,ti,kw OR Gambia*:ab,ti,kw OR 'Georgia

(republic)'/exp OR Georgia*:ab,ti,kw OR Ghana*:ab,ti,kw OR Grenada*:ab,ti,kw OR Guadeloupe:ab,ti,kw OR Guatemala*:ab,ti,kw OR Guian*:ab,ti,kw OR Guinea*:ab,ti,kw OR Guyan*:ab,ti,kw OR Haiti*:ab,ti,kw OR Hondura*:ab,ti,kw OR India:ab,ti,kw OR Indian*:ab,ti,kw OR Indonesia*:ab,ti,kw OR Iran*:ab,ti,kw OR Iraq*:ab,ti,kw OR Jamaica*:ab,ti,kw OR Jordan*:ab,ti,kw OR Kazakh*:ab,ti,kw OR Kenya*:ab,ti,kw OR Kiribati:ab,ti,kw OR 'people`s republic of korea':ab,ti,kw OR @North Korea@:ab,ti,kw OR 'Kosovo'/exp OR Kosovo:ab,ti,kw OR 'Kosovar'/exp OR Kosovar*:ab,ti,kw OR Kyrgyz*:ab,ti,kw OR Lao:ab,ti,kw OR Laos:ab,ti,kw OR Laotian*:ab,ti,kw OR Lebanon:ab,ti,kw OR Lebanes*:ab,ti,kw OR Lesotho:ab,ti,kw OR Liberia*:ab,ti,kw OR Libya*:ab,ti,kw OR 'Macedonia (republic)'/exp OR Macedonia*:ab,ti,kw OR Madagascar*:ab,ti,kw OR Malawi*:ab,ti,kw OR Malvinas:ab,ti,kw OR Malaysia*:ab,ti,kw OR 'Maldives'/exp OR Maldives:ab,ti,kw OR Mali:ab,ti,kw OR @Marshall Island*@:ab,ti,kw OR Mauritania*:ab,ti,kw OR 'Mauritius'/exp OR Mauriti*:ab,ti,kw OR Mayotte:ab,ti,kw OR 'Mexico'/exp OR 'Mexican'/exp OR Mexican*:ab,ti,kw OR Micronesia*:ab,ti,kw OR Moldova*:ab,ti,kw OR Mongolia*:ab,ti,kw OR 'Montenegro (republic)'/exp OR Montenegr*:ab,ti,kw OR Montserrat:ab,ti,kw OR Morocc*:ab,ti,kw OR Mozambique:ab,ti,kw OR Myanmar:ab,ti,kw OR Burmese*:ab,ti,kw OR Burma:ab,ti,kw OR Namibia*:ab,ti,kw OR Nauru:ab,ti,kw OR Nepal*:ab,ti,kw OR @Netherlands Antilles@:ab,ti,kw OR Nicaragua*:ab,ti,kw OR Niger*:ab,ti,kw OR Niue:ab,ti,kw OR Pakistan*:ab,ti,kw OR Paraguay*:ab,ti,kw OR Peru*:ab,ti,kw OR Philippin*:ab,ti,kw OR Pitcairn:ab,ti,kw OR 'Romania'/exp OR Romania*:ab,ti,kw OR Rwanda*:ab,ti,kw OR @Sao Tome@:ab,ti,kw OR Principe:ab,ti,kw OR Senegal*:ab,ti,kw OR 'Serbia'/exp OR Serbia*:ab,ti,kw OR @Sierra Leone*@:ab,ti,kw OR @Solomon Island*@:ab,ti,kw OR Somalia*:ab,ti,kw OR @South Africa*@:ab,ti,kw OR @Sri Lanka@:ab,ti,kw OR @St Helena@:ab,ti,kw OR @Saint Helena@:ab,ti,kw OR @St Lucia@:ab,ti,kw OR @Saint Lucia@:ab,ti,kw OR @St Vincent@:ab,ti,kw OR @Saint Vincent@:ab,ti,kw OR Grenad*:ab,ti,kw OR Sudan*:ab,ti,kw OR Suriname*:ab,ti,kw OR Swaziland*:ab,ti,kw OR Eswatini*:ab,ti,kw OR Syria*:ab,ti,kw OR Tajik*:ab,ti,kw OR Tanzania*:ab,ti,kw OR Thai*:ab,ti,kw OR Timor*:ab,ti,kw OR Togo*:ab,ti,kw OR Tokelau:ab,ti,kw OR Tonga*:ab,ti,kw OR Tunisia*:ab,ti,kw OR Turkey:ab,ti,kw OR Turkish:ab,ti,kw OR Turkmen*:ab,ti,kw OR Tuvalu*:ab,ti,kw OR Uganda*:ab,ti,kw OR Ukrain*:ab,ti,kw OR Uzbeki*:ab,ti,kw OR Vanuatu*:ab,ti,kw OR Venezuela*:ab,ti,kw OR Vietnam*:ab,ti,kw OR @Viet nam*@:ab,ti,kw OR @West Bank@:ab,ti,kw OR Gaza*:ab,ti,kw OR Palestin*:ab,ti,kw OR @Wallis and Futuna@:ab,ti,kw OR Yemen*:ab,ti,kw OR Zambia*:ab,ti,kw OR Zimbabw*:ab,ti,kw OR @Western Sahara@:ab,ti,kw OR Argentin*:ab,ti,kw)
AND
('follow up studies'/exp OR 'prospective studies'/exp OR 'Follow Up':ab,ti,kw OR random*:ab,ti,kw OR trial*:ab,ti,kw)

Web of Science Core Collection(n=1,658)
AB=("iron" OR "Ferric" OR "Ferrous" OR "Ferric Compounds" OR " Ferrous Compounds")
AND
AB=("adolescence" OR "adolescent" OR "adolescents" OR "boy" OR "boys" OR "girl" OR "girls" OR "pre pubert*" OR "pre teen*" OR "preadolescen*" OR "prepubert*" OR "prepubescen*" OR "preteen*" OR "puberty" OR "pubescen*" OR "school age*" OR "schoolage*" OR "teen" OR "teenage*" OR "teens" OR "age 5" OR "age 6" OR "age 7" OR "age 8" OR "age 9" OR "age 10" OR

"age 11" OR "age 12" OR "age 13" OR "age 14" OR "age 15" OR "age 16" OR "age 17" OR "age 18" OR "age 19" OR "aged 5" OR "aged 6" OR "aged 7" OR "aged 8" OR "aged 9" OR "aged 10" OR "aged 11" OR "aged 12" OR "aged 13" OR "aged 14" OR "aged 15" OR "aged 16" OR "aged 17" OR "aged 18" OR "aged 19" OR "5 years old" OR "6 years old" OR "7 years old" OR "8 years old" OR "9 years old" OR "10 years old" OR "11 years old" OR "12 years old" OR "13 years old" OR "14 years old" OR "15 years old" OR "16 years old" OR "17 years old" OR "18 years old" OR "19 years old" OR "5 years of age" OR "6 years of age" OR "7 years of age" OR "8 years of age" OR "9 years of age" OR "10 years of age" OR "11 years of age" OR "12 years of age" OR "13 years of age" OR "14 years of age" OR "15 years of age" OR "16 years of age" OR "17 years of age" OR "18 years of age" OR "19 years of age" OR "child" OR "children" OR "student" OR "child" OR "children" OR "school children" OR "kids" OR "kid" OR "student*" OR "teen*" OR "adolescen*" OR "preteen*")
AND
AB=("developing countr*" OR "developing nation*" OR "less developed countr*" OR "less developed nation*" OR "least developed countr*" OR "least developed nation*" OR "third world nation*" OR "third world countr*" OR "under developed nation*" OR "underdeveloped nation*" OR "under developed countr*" OR "underdeveloped countr*" OR "middle income countr*" OR "middle income nation*" OR "low income countr*" OR "low income nation*" OR "poor countr*" OR "poor nation*" OR "lmic" OR "lmics" OR "africa" OR "asia" OR "south america*" OR "latin america*" OR "central america*" OR "Afghanistan*" OR "Albania*" OR "Algeria*" OR "Samoa*" OR "Angola*" OR "Armenia*" OR "Azerbaijan*" OR "Bangladesh*" OR "Bengali" OR "Belarus*" OR "Belize" OR "Benin" OR "Bhutan*" OR "Bolivia*" OR "Bosnia*" OR "Herzegovina*" OR "Botswana*" OR "Brazil*" OR "Bulgaria*" OR "Burkina Faso" OR "Burundi*" OR "Cabo Verd*" OR "Cape Verd*" OR "Cambodia*" OR "Cameroon*" OR "Central African*" OR "Chad*" OR "China" OR "Chinese" OR "Colombia*" OR "Comoros" OR "Congo" OR "Cook Islands" OR "Costa Rica*" OR "Cote d'Ivoire" OR "Ivory Coast" OR "Cuba" OR "Cuban" OR "Djibouti" OR "Dominica*" OR "Ecuador" OR "Egypt" OR "El Salvador*" OR "Eritrea*" OR "Ethiopia*" OR "Falkland Islands" OR "Fiji*" OR "Gabon*" OR "Gambia*" OR "Georgia*" OR "Ghana*" OR "Grenada*" OR "Guadeloupe" OR "Guatemala*" OR "Guian*" OR "Guinea*" OR "Guyan*" OR "Haiti*" OR "Hondura*" OR "India" OR "Indian*" OR "Indonesia*" OR "Iran*" OR "Iraq*" OR "Jamaica*" OR "Jordan*" OR "Kazakh*" OR "Kenya*" OR "Kiribati" OR "People's Republic of Korea" OR "North Korea" OR "Kosovo" OR "Kosovar*" OR "Kyrgyz*" OR "Lao" OR "Laos" OR "Laotian*" OR "Lebanon" OR "Lebanes*" OR "Lesotho" OR "Liberia*" OR "Libya*" OR "Macedonia*" OR "Madagascar*" OR "Malawi*" OR "Malvinas" OR "Malaysia*" OR "Maldives" OR "Mali" OR "Marshall Island*" OR "Mauritania*" OR "Mauriti*" OR "Mayotte" OR "Mexico" OR "Mexican*" OR "Micronesia*" OR "Moldova*" OR "Mongolia*" OR "Montenegr*" OR "Montserrat" OR "Morocc*" OR "Mozambique" OR "Myanmar" OR "Burmese*" OR "Burma" OR "Namibia*" OR "Nauru" OR "Nepal*" OR "Netherlands Antilles" OR "Nicaragua*" OR "Niger*" OR "Niue" OR "Pakistan*" OR "Paraguay*" OR "Peru*" OR "Philippin*" OR "Pitcairn" OR "Romania*" OR "Rwanda*" OR "Sao Tome" OR "Principe" OR "Senegal*" OR "Serbia*" OR "Sierra Leone*" OR "Solomon Island*" OR "Somalia*" OR "South Africa*" OR "Sri Lanka" OR "St Helena" OR "Saint Helena" OR "St Lucia" OR "Saint Lucia" OR "St Vincent" OR "Saint Vincent" OR "Grenad*" OR "Sudan*" OR "Suriname*" OR "Swaziland*" OR "Eswatini*" OR "Syria*" OR "Tajik*" OR "Tanzania*" OR "Thai*" OR "Timor*" OR "Togo*" OR "Tokelau" OR "Tonga*" OR

"Tunisia*" OR "Turkey" OR "Turkish" OR "Turkmen*" OR "Tuvalu*" OR "Uganda*" OR "Ukrain*" OR "Uzbeki*" OR "Vanuatu*" OR "Venezuela*" OR "Vietnam*" OR "Viet nam*" OR "West Bank" OR "Gaza*" OR "Palestin*" OR "Wallis and Futuna" OR "Yemen*" OR "Zambia*" OR "Zimbabw*" OR "Western Sahara" OR "Argentin*" OR "Gambia*" OR "Georgia*" OR "Ghana*" OR "Grenada*" OR "Guadeloupe" OR "Guatemala*" OR "Guian*" OR "Guinea*" OR "Guyan*" OR "Haiti*" OR "Hondura*" OR "India" OR "Indian*" OR "Indonesia*" OR "Iran*" OR "Iraq*" OR "Jamaica*" OR "Jordan*" OR "Kazakh*" OR "Kenya*" OR "Kiribati" OR "People's Republic of Korea" OR "North Korea" OR "Kosovo" OR "Kosovar*" OR "Kyrgyz*" OR "Lao" OR "Laos" OR "Laotian*" OR "Lebanon" OR "Lebanes*" OR "Lesotho" OR "Liberia*" OR "Libya*" OR "Macedonia*" OR "Madagascar*" OR "Malawi*" OR "Malvinas" OR "Malaysia*" OR "Maldives" OR "Mali" OR "Marshall Island*" OR "Mauritania*" OR "Mauriti*" OR "Mayotte" OR "Mexico" OR "Mexican*" OR "Micronesia*" OR "Moldova*" OR "Mongolia*" OR "Montenegr*" OR "Montserrat" OR "Morocc*" OR "Mozambique" OR "Myanmar" OR "Burmese*" OR "Burma" OR "Namibia*" OR "Nauru" OR "Nepal*" OR "Netherlands Antilles" OR "Nicaragua*" OR "Niger*" OR "Niue" OR "Pakistan*" OR "Paraguay*" OR "Peru*")
AND
AB=("controlled clinical trial" OR "randomized controlled trial" OR "clinical trial" OR "Follow Up" OR "random*" OR "trial*")

CINAHL(n=857):
(MH "Iron" OR MH "Ferric Compounds" OR MH "Ferrous Compounds" OR TI iron OR TI "Ferric" OR TI "Ferrous" OR TI "Ferric Compounds" OR TI "Ferrous Compounds" OR AB iron OR AB "Ferric" OR AB "Ferrous" OR AB "Ferric Compounds" OR AB "Ferrous Compounds")
AND
(MH "Child+" OR MH "Students+" OR MH "Adolescence+" OR TI adolescence OR TI adolescent OR TI adolescents OR TI boy OR TI boys OR TI girl OR TI girls OR TI pre pubert* OR TI pre teen* OR TI preadolescen* OR TI prepubert* OR TI prepubescen* OR TI preteen* OR TI puberty OR TI pubescen* OR TI school age* OR TI schoolage* OR TI teen OR TI teenage* OR TI teens OR TI age 5 OR TI age 6 OR TI age 7 OR TI age 8 OR TI age 9 OR TI age 10 OR TI age 11 OR TI age 12 OR TI age 13 OR TI age 14 OR TI age 15 OR TI age 16 OR TI age 17 OR TI age 18 OR TI age 19 OR TI aged 5 OR TI aged 6 OR TI aged 7 OR TI aged 8 OR TI aged 9 OR TI aged 10 OR TI aged 11 OR TI aged 12 OR TI aged 13 OR TI aged 14 OR TI aged 15 OR TI aged 16 OR TI aged 17 OR TI aged 18 OR TI aged 19 OR TI 5 years old OR TI 6 years old OR TI 7 years old OR TI 8 years old OR TI 9 years old OR TI 10 years old OR TI 11 years old OR TI 12 years old OR TI 13 years old OR TI 14 years old OR TI 15 years old OR TI 16 years old OR TI 17 years old OR TI 18 years old OR TI 19 years old OR TI 5 years of age OR TI 6 years of age OR TI 7 years of age OR TI 8 years of age OR TI 9 years of age OR TI 10 years of age OR TI 11 years of age OR TI 12 years of age OR TI 13 years of age OR TI 14 years of age OR TI 15 years of age OR TI 16 years of age OR TI 17 years of age OR TI 18 years of age OR TI 19 years of age OR TI child OR TI children OR TI child OR TI children OR TI schoolchildren OR TI kids OR TI kid OR TI student* OR TI teen* OR TI adolescen* OR TI preteen* OR AB adolescence OR AB adolescent OR AB adolescents OR AB boy OR AB boys OR AB girl OR AB girls OR AB pre pubert* OR AB pre teen* OR AB preadolescen* OR AB prepubert* OR AB prepubescen* OR AB preteen* OR AB puberty OR AB pubescen* OR AB school age* OR AB schoolage* OR AB teen OR AB

teenage* OR AB teens OR AB age 5 OR AB age 6 OR AB age 7 OR AB age 8 OR AB age 9 OR AB age 10 OR AB age 11 OR AB age 12 OR AB age 13 OR AB age 14 OR AB age 15 OR AB age 16 OR AB age 17 OR AB age 18 OR AB age 19 OR AB aged 5 OR AB aged 6 OR AB aged 7 OR AB aged 8 OR AB aged 9 AB aged 10 OR AB aged 11 OR AB aged 12 OR AB aged 13 OR AB aged 14 OR AB aged 15 OR AB aged 16 OR AB aged 17 OR AB aged 18 OR AB aged 19 OR AB 10 years old OR AB 11 years old OR AB 12 years old OR AB 13 years old OR AB 14 years old OR AB 15 years old OR AB 16 years old OR AB 17 years old OR AB 18 years old OR AB 19 years old OR AB 10 years of age OR AB 11 years of age OR AB 12 years of age OR AB 13 years of age OR AB 14 years of age OR AB 15 years of age OR AB 16 years of age OR AB 17 years of age OR AB 18 years of age OR AB 19 years of age OR AB child OR AB children OR AB child OR AB children OR AB schoolchildren OR AB kids OR AB kid OR AB student* OR AB teen* OR AB adolescen* OR AB preteen*)
AND
(MH "Developing Countries" OR TI developing countr* OR TI developing nation* OR TI less developed countr* OR TI less developed nation* OR TI least developed countr* OR TI least developed nation* OR TI third world nation* OR TI third world countr* OR TI under developed nation* OR TI underdeveloped nation* OR TI under developed countr* OR TI underdeveloped countr* OR TI middle income countr* OR TI middle income nation* OR TI low income countr* OR TI low income nation* OR TI poOR TI countr* OR TI poOR TI nation* OR TI lmic OR TI lmics OR MH "Africa+" OR MH "Asia+" OR MH "South America+" OR MH "Latin America" OR MH "Central America+" OR TI africa OR TI asia OR TI south america* OR TI latin america* OR TI central america* OR TI Afghanistan* OR TI Albania* OR TI Algeria* OR TI Samoa* OR TI Angola* OR TI Armenia* OR TI Azerbaijan* OR TI Bangladesh* OR TI Bengali OR TI Belarus* OR TI Belize OR TI Benin OR TI Bhutan* OR TI Bolivia* OR TI Bosnia* OR TI Herzegovina* OR TI Botswana* OR TI Brazil* OR TI Bulgaria* OR TI Burkina Faso OR TI Burundi* OR TI Cabo Verd* OR TI Cape Verd* OR TI Cambodia* OR TI Cameroon* OR TI Central African* OR TI Chad* OR TI China OR TI Chinese OR TI Colombia* OR TI Comoros OR TI Congo OR TI Cook Islands OR TI Costa Rica* OR TI Cote d'Ivoire OR TI Ivory Coast OR TI Cuba OR TI Cuban OR TI Djibouti OR TI Dominica* OR TI Ecuador OR TI Egypt OR TI El Salvador* OR TI Eritrea* OR TI Ethiopia* OR TI Falkland Islands OR TI Fiji* OR TI Gabon* OR TI Gambia* OR TI Georgia* OR TI Ghana* OR TI Grenada* OR TI Guadeloupe OR TI Guatemala* OR TI Guian* OR TI Guinea* OR TI Guyan* OR TI Haiti* OR TI Hondura* OR TI India OR TI Indian* OR TI Indonesia* OR TI Iran* OR TI Iraq* OR TI Jamaica* OR TI Jordan* OR TI Kazakh* OR TI Kenya* OR TI Kiribati OR TI People's Republic of Korea OR TI North Korea OR TI Kosovo OR TI Kosovar* OR TI Kyrgyz* OR TI Lao OR TI Laos OR TI Laotian* OR TI Lebanon OR TI Lebanes* OR TI Lesotho OR TI Liberia* OR TI Libya* OR TI Macedonia* OR TI Madagascar* OR TI Malawi* OR TI Malvinas OR TI Malaysia* OR TI Maldives OR TI Mali OR TI Marshall Island* OR TI Mauritania* OR TI Mauriti* OR TI Mayotte OR MH Mexico OR TI Mexican* OR TI Micronesia* OR TI Moldova* OR TI Mongolia* OR TI Montenegr* OR TI Montserrat OR TI Morocc* OR TI Mozambique OR TI Myanmar OR TI Burmese* OR TI Burma OR TI Namibia* OR TI Nauru OR TI Nepal* OR TI Netherlands Antilles OR TI Nicaragua* OR TI Niger* OR TI Niue OR TI Pakistan* OR TI Paraguay* OR TI Peru* OR TI Philippin* OR TI Pitcairn OR TI Romania* OR TI Rwanda* OR TI Sao Tome OR TI Principe OR TI Senegal* OR TI Serbia* OR TI Sierra Leone* OR TI Solomon Island* OR TI Somalia* OR TI South Africa* OR TI Sri Lanka

OR TI St Helena OR TI Saint Helena OR TI St Lucia OR TI Saint Lucia OR TI St Vincent OR TI Saint Vincent OR TI Grenad* OR TI Sudan* OR TI Suriname* OR TI Swaziland* OR TI Eswatini* OR TI Syria* OR TI Tajik* OR TI Tanzania* OR TI Thai* OR TI Timor* OR TI Togo* OR TI Tokelau OR TI Tonga* OR TI Tunisia* OR TI Turkey OR TI Turkish OR TI Turkmen* OR TI Tuvalu* OR TI Uganda* OR TI Ukrain* OR TI Uzbeki* OR TI Vanuatu* OR TI Venezuela* OR TI Vietnam* OR TI Viet nam* OR TI West Bank OR TI Gaza* OR TI Palestin* OR TI Wallis and Futuna OR TI Yemen* OR TI Zambia* OR TI Zimbabw* OR TI Western Sahara OR TI Argentin* OR AB developing countr* OR AB developing nation* OR AB less developed countr* OR AB less developed nation* OR AB least developed countr* OR AB least developed nation* OR AB third world nation* OR AB third world countr* OR AB under developed nation* OR AB underdeveloped nation* OR AB under developed countr* OR AB underdeveloped countr* OR AB middle income countr* OR AB middle income nation* OR AB low income countr* OR AB low income nation* OR AB poOR AB countr* OR AB poOR AB nation* OR AB lmic OR AB lmics OR AB africa OR AB asia OR AB south america* OR AB latin america* OR AB central america* OR AB Afghanistan* OR AB Albania* OR AB Algeria* OR AB Samoa* OR AB Angola* OR AB Armenia* OR AB Azerbaijan* OR AB Bangladesh* OR AB Bengali OR AB Belarus* OR AB Belize OR AB Benin OR AB Bhutan* OR AB Bolivia* OR AB Bosnia* OR AB Herzegovina* OR AB Botswana* OR AB Brazil* OR AB Bulgaria* OR AB Burkina Faso OR AB Burundi* OR AB Cabo Verd* OR AB Cape Verd* OR AB Cambodia* OR AB Cameroon* OR AB Central African* OR AB Chad* OR AB China OR AB Chinese OR AB Colombia* OR AB Comoros OR AB Congo OR AB Cook Islands OR AB Costa Rica* OR AB Cote d'Ivoire OR AB Ivory Coast OR AB Cuba OR AB Cuban OR AB DjibouAB OR AB Dominica* OR AB Ecuador OR AB Egypt OR AB El Salvador* OR AB Eritrea* OR AB Ethiopia* OR AB Falkland Islands OR AB Fiji* OR AB Gabon* OR AB Gambia* OR AB Georgia* OR AB Ghana* OR AB Grenada* OR AB Guadeloupe OR AB Guatemala* OR AB Guian* OR AB Guinea* OR AB Guyan* OR AB Haiti* OR AB Hondura* OR AB India OR AB Indian* OR AB Indonesia* OR AB Iran* OR AB Iraq* OR AB Jamaica* OR AB Jordan* OR AB Kazakh* OR AB Kenya* OR AB KiribaAB OR AB People's Republic of Korea OR AB North Korea OR AB Kosovo OR AB Kosovar* OR AB Kyrgyz* OR AB Lao OR AB Laos OR AB Laotian* OR AB Lebanon OR AB Lebanes* OR AB Lesotho OR AB Liberia* OR AB Libya* OR AB Macedonia* OR AB Madagascar* OR AB Malawi* OR AB Malvinas OR AB Malaysia* OR AB Maldives OR AB Mali OR AB Marshall Island* OR AB Mauritania* OR AB Mauriti* OR AB Mayotte OR AB Mexican* OR AB Micronesia* OR AB Moldova* OR AB Mongolia* OR AB Montenegr* OR AB Montserrat OR AB Morocc* OR AB Mozambique OR AB Myanmar OR AB Burmese* OR AB Burma OR AB Namibia* OR AB Nauru OR AB Nepal* OR AB Netherlands Antilles OR AB Nicaragua* OR AB Niger* OR AB Niue OR AB Pakistan* OR AB Paraguay* OR AB Peru* OR AB Philippin* OR AB Pitcairn OR AB Romania* OR AB Rwanda* OR AB Sao Tome OR AB Principe OR AB Senegal* OR AB Serbia* OR AB Sierra Leone* OR AB Solomon Island* OR AB Somalia* OR AB South Africa* OR AB Sri Lanka OR AB St Helena OR AB Saint Helena OR AB St Lucia OR AB Saint Lucia OR AB St Vincent OR AB Saint Vincent OR AB Grenad* OR AB Sudan* OR AB Suriname* OR AB Swaziland* OR AB Eswatini* OR AB Syria* OR AB Tajik* OR AB Tanzania* OR AB Thai* OR AB Timor* OR AB Togo* OR AB Tokelau OR AB Tonga* OR AB Tunisia* OR AB Turkey OR AB Turkish OR AB Turkmen* OR AB Tuvalu* OR AB Uganda* OR AB Ukrain* OR AB Uzbeki* OR AB Vanuatu* OR AB Venezuela* OR AB Vietnam* OR AB Viet nam* OR AB West Bank OR AB

Gaza* OR AB Palestin* OR AB Wallis and Futuna OR AB Yemen* OR AB Zambia* OR AB Zimbabw* OR AB Western Sahara OR AB Argentin*)

AND

(TI "controlled clinical trial" OR TI "randomized controlled trial" OR TI "clinical trial" OR TI "Follow Up" OR TI random* OR TI trial* OR AB "controlled clinical trial" OR AB "randomized controlled trial" OR AB "clinical trial" OR AB "Follow Up" OR AB random* OR AB trial*)

Supplementary Text S2. Definition of cognitive function indicators

The names of the indicators of cognitive function are different, we classify them according to the results of expert consultation as follows:

**Cogitative function**	**Indicators**
Intelligence	Kaufman assessment battery for children-simultaneous processing: Triangles[1], Weschler adult intelligence scale[2], the raven-colored progressive matrices [3], mazes[4], IQ [5,6] the test of nonverbal intelligence[7]
Attention	Digit span[4]
Short-term memory	Kaufman assessment battery for children-sequential processing: Hand movement[1], short term memory[2], visual recall[4]
Long-term memory	Kaufman assessment battery for children-learning abilities: Atlantis delayed[1], long term memory[2]
School Performance	Scholastic performance test[2], math[3,5,7]

Supplementary Text S3. Data conversion and combine the study arms

In this study, all the original data had the data of baseline and endpoint values, and the results reported as Mean ± SE were converted into SD by the following formula[8]:

Data relate to	Core statistics	Related, commonly-reported statistics
Intervention E	N, M_E, SD_E	Standard error of M_E.
Intervention C	N, M_C, SD_C	Standard error of M_C.
Difference between E and C	N, MD, SD_{diff}	Standard error of MD;<br>Confidence interval for MD;<br>Paired t-statistic;<br>P value from paired t-test.

$$SE(MD) = \frac{SD_{diff}}{\sqrt{N}}.$$

Then, we calculate the SD value of the change value using the following formula[8]:

$$SD_{diff} = \sqrt{SD_E^2 + SD_C^2 - (2 \times Corr \times SD_E \times SD_C)}.$$

In this study, we assumed a correlation coefficient of 0.5[9].

When a study reported data into subgroups, we combined the subgroups into a single intervention group by the following formula [3].

	Group 1 (e.g. males)	Group 2 (e.g. females)	Combined groups
**Sample size**	N_1	N_2	$N_1 + N_2$
**Mean**	M_1	M_2	$\frac{N_1M_1 + N_2M_2}{N_1 + N_2}$
**SD**	SD_1	SD_2	$\sqrt{\frac{(N_1-1)SD_1^2 + (N_2-1)SD_2^2 + \frac{N_1N_2}{N_1+N_2}(M_1^2 + M_2^2 - 2M_1M_2)}{N_1 + N_2 - 1}}$

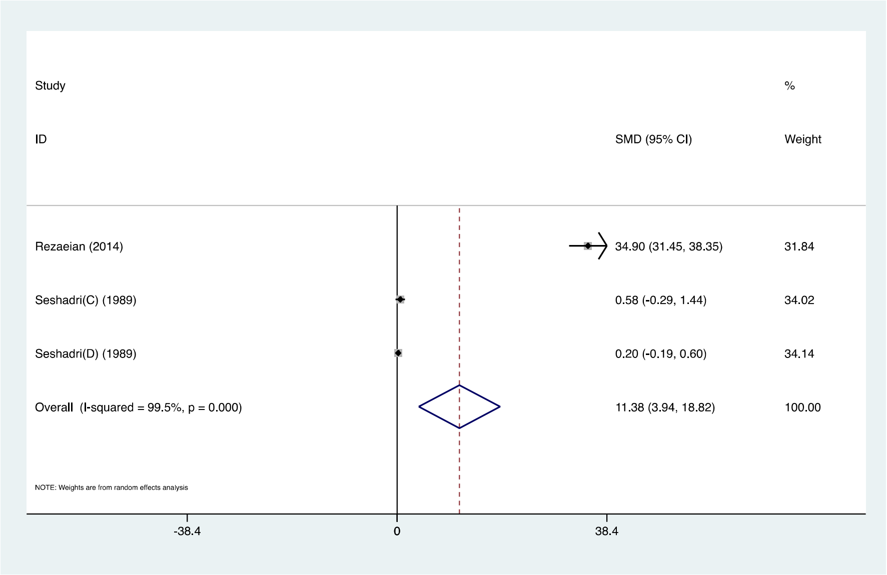

Supplementary Figure S1. Forest plot of the effect of iron supplementation on attention in children and adolescents

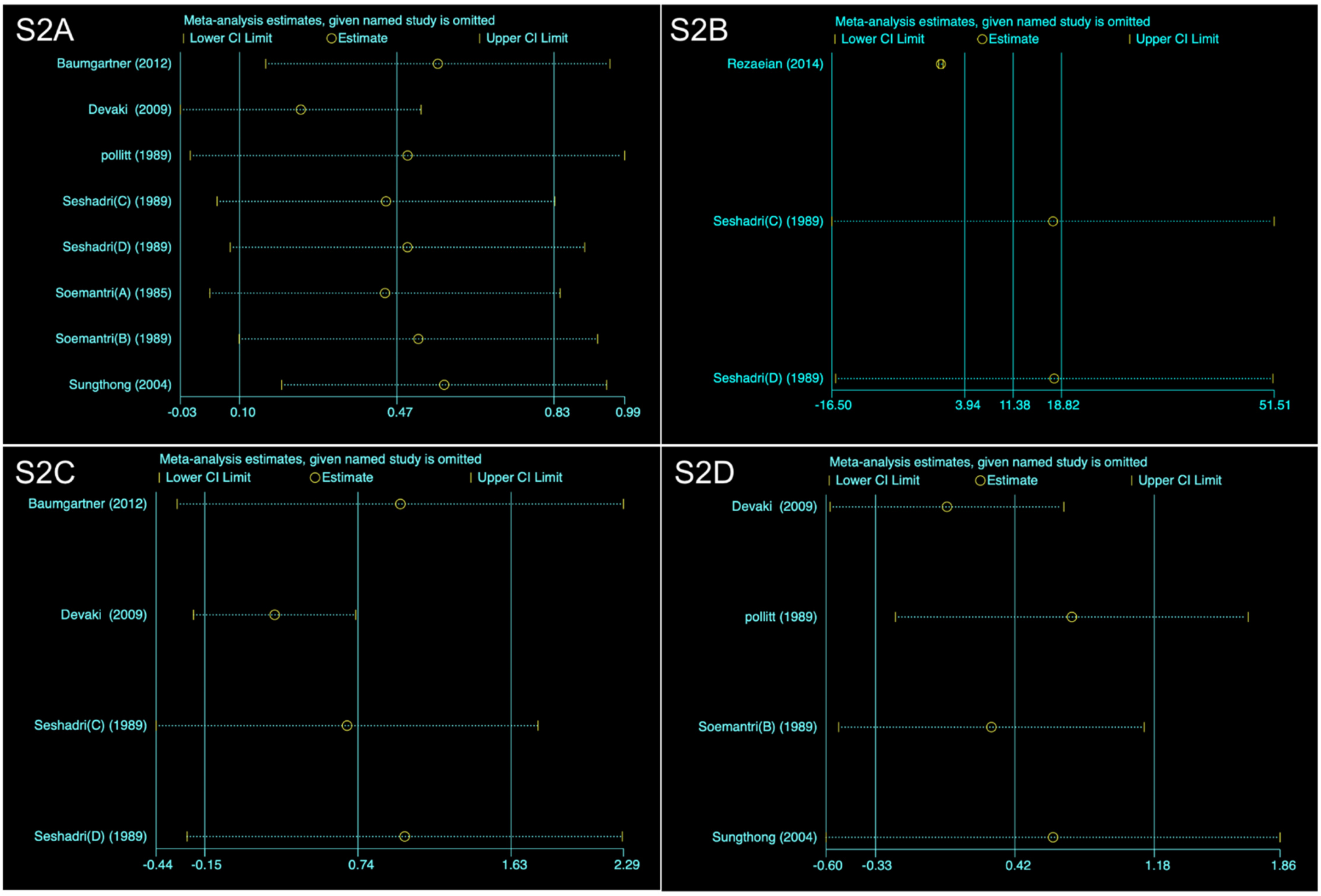

Supplementary Figure S2. Sensitive analysis of cognitive function(a) Sensitive analysis of the effect of iron supplementation on intelligence in older children and young adolescents. (b) Sensitive analysis of the effect of iron supplementation on attention in older children and young adolescents. (c) Sensitive analysis of the effect of iron supplementation on short-term memory in older children and young adolescents. (d) Sensitive analysis of the effect of iron supplementation on school performance in older children and young adolescents.

Supplementary Figure S3. Risk of bias of included studies.

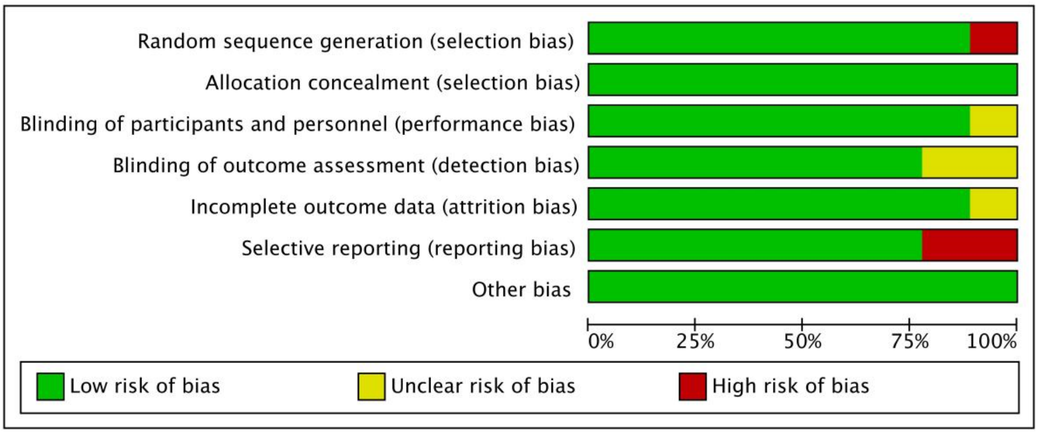

Supplementary Figure S3a. Risk of bias graph of included studies.

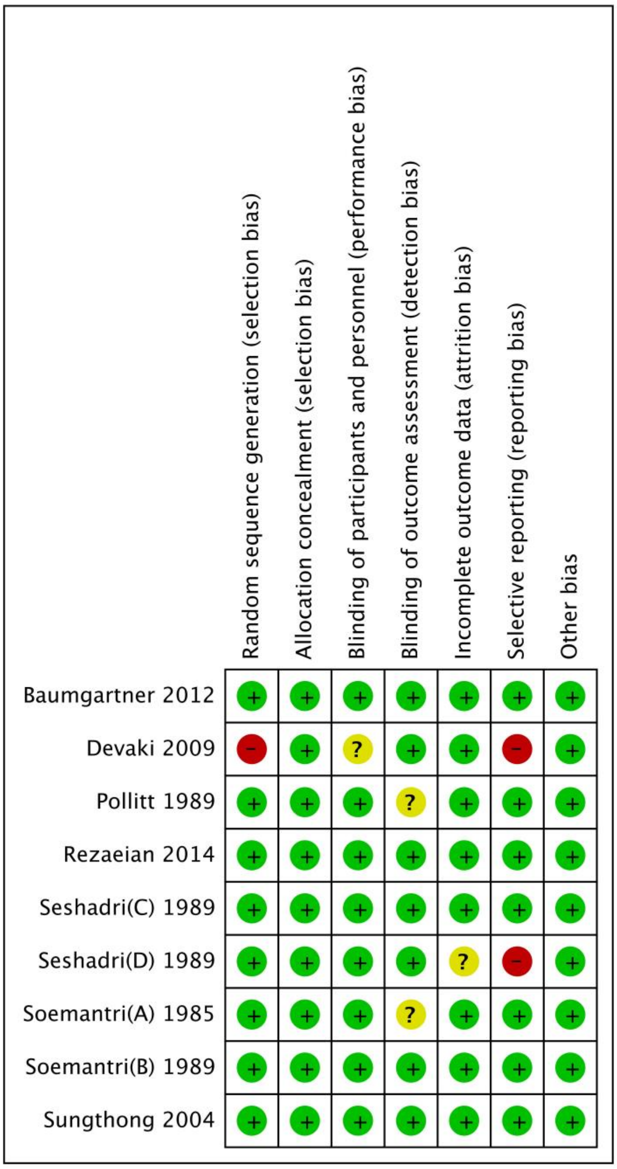

Supplementary Figure S3b. Risk of bias summary of included studies.

Reference:

1. Baumgartner, J.; Smuts, C.M.; Malan, L.; Kvalsvig, J.; van Stuijvenberg, M.E.; Hurrell, R.F.; Zimmermann, M.B. Effects of iron and n-3 fatty acid supplementation, alone and in combination, on cognition in school children: a randomized, double-blind, placebo-controlled intervention in South Africa. *The American journal of clinical nutrition* **2012**, *96*, 1327-1338.
2. Devaki, P.B.; Chandra, R.K.; Geisser, P. Effects of oral iron(III) hydroxide polymaltose complex supplementation on hemoglobin increase, cognitive function, affective behavior and scholastic performance of adolescents with varying iron status: a single centre prospective placebo controlled study. *Arzneimittelforschung* **2009**, *59*, 303-310, doi:10.1055/s-0031-1296401.
3. Pollitt, E.; Hathirat, P.; Kotchabhakdi, N.J.; Missell, L.; Valyasevi, A. Iron deficiency and educational achievement in Thailand. *Am J Clin Nutr* **1989**, *50*, 687-696; discussion 696-687, doi:10.1093/ajcn/50.3.687.
4. Seshadri, S.; Gopaldas, T. Impact of iron supplementation on cognitive functions in preschool and school-aged children: the Indian experience. *The American Journal of Clinical Nutrition* **1989**, *50*, 675-686.
5. Soemantri, A. Preliminary findings on iron supplementation and learning achievement of rural Indonesian children. *The American Journal of Clinical Nutrition* **1989**, *50*, 698-702.
6. Soemantri, A.; Pollitt, E.; Kim, I. Iron deficiency anemia and educational achievement. *The American journal of clinical nutrition* **1985**, *42*, 1221-1228.
7. Sungthong, R.; Mo-Suwan, L.; Chongsuvivatwong, V.; Geater, A.F. Once-weekly and 5-days a week iron supplementation differentially affect cognitive function but not school performance in Thai children. *The Journal of nutrition* **2004**, *134*, 2349-2354.
8. Higgins, J.; Deeks, J.; Altman, D.G. Chapter 16: Special topics in statistics. **2011**.
9. Sachdev, H.; Gera, T.; Nestel, P.J.P.H.N. Effect of iron supplementation on mental and motor development in children: systematic review of randomised controlled trials. **2005**.
